# Supplementary material for: Causal Relationship between Aging and Anorexia Nervosa: A White-Matter-Microstructure-Mediated Mendelian Randomization Analysis
Source: Biomedicines. 2024 Aug 16;12(8):1874. doi: 10.3390/biomedicines12081874 (PMC11351342; doi:10.3390/biomedicines12081874)
Supplement: Supplementary file 1 [file biomedicines-12-01874-s001.zip › Supplementary Figure/Figure S8/Leaveoneout plot of MR analysis for TL to ubm-b-1551.pdf]

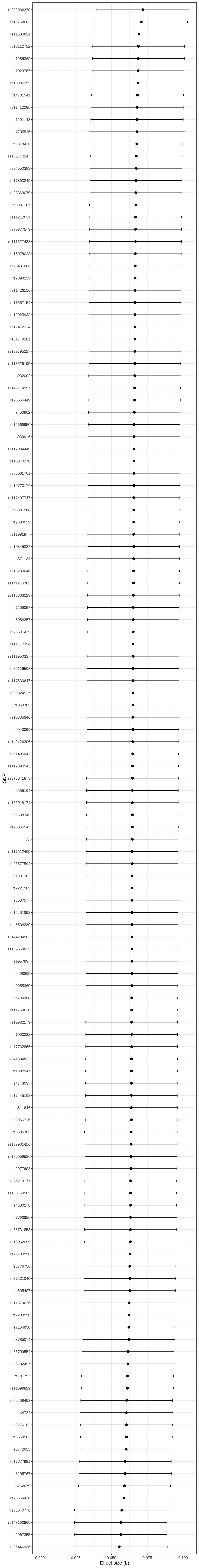

rs202034370
rs10769683
rs115848211
rs10112752
rs1985369
rs1023767
rs10905255
rs4731541
rs11412296
rs1291143
rs7705526
rs9878436
rs185174247
rs34550383
rs17803849
rs28363070
rs3891167
rs11212631
rs79977579
rs111527438
rs16978028
rs78491606
rs7099229
rs11426156
rs11557154
rs12925933
rs12412214
rs61748181
rs139795227
rs113525195
rs932002
rs145114957
rs76666449
rs450962
rs12369950
rs939916
rs117034449
rs10840270
rs56061761
rs10773176
rs117407747
rs6881568
rs9600019
rs11991877
rs10845387
rs871134
rs13230646
rs141214782
rs116863223
rs7209057
rs6054257
rs73581419
rs11117354
rs111950327
rs80116508
rs117630647
rs80324517
rs869785
rs10805346
rs9940099
rs142426306
rs61405042
rs112394943
rs376641875
rs2555104
rs188918174
rs2538745
rs76065543
All
rs117512405
rs28577594
rs1907702
rs7221585
rs6587577
rs12451892
rs45604339
rs144204502
rs139669835
rs1957937
rs4498805
rs9955360
rs6790988
rs11769630
rs12932179
rs1003322
rs77732866
rs41304832
rs1332941
rs4743037
rs17445108
rs611646
rs2056726
rs6536702
rs137901416
rs182059586
rs2977608
rs76219171
rs150150565
rs4530278
rs7790856
rs66731853
rs13062095
rs73730598
rs6776756
rs77231040
rs4695407
rs11579626
rs2230590
rs7164950
rs3785074
rs56799554
rs8102497
rs131797
rs11699829
rs59409453
rs4724
rs2276182
rs6669563
rs5742915
rs17677991
rs8105767
rs762679
rs75664430
rs35640778
rs143190905
rs2967355
rs35446936
SNP
0.000
0.025
0.050
0.075
0.100
Effect size (b)
